# Provision of Temporary Access to Inpatient Hemodialysis to Uninsured Patients Initiating Hemodialysis

**DOI:** 10.1001/jamanetworkopen.2025.44295

**Published:** 2025-11-18

**Authors:** Josh Banerjee, Hugh Gordon, Victoria E. Walsh, Jasmine S. Espiritu, Catherine Canamar, Soodtida Tangprahaphorn, Hannah H. Oh, Jacklyn P. Nguyen, Tammy Yun, Young Shin Seo, Young Song, Mark Redulla, Melissa Alvarez, Arshia Ghaffari, Douglass Hutcheon, Jan Shoenberger, Michael Varnal, Nancy Blake, Charles E. Coffey, Brad Spellberg

**Affiliations:** 1 Hospital Administration, Los Angeles General Medical Center, Los Angeles, California; 2 Department of Regulatory Affairs, Los Angeles General Medical Center, Los Angeles, California; 3 Department of Quality, Los Angeles General Medical Center, Los Angeles, California; 4 Division of Hemodialysis, Department of Nursing, Los Angeles General Medical Center, Los Angeles, California; 5 Department of Medicine, Keck School of Medicine at the University of Southern California, Los Angeles; 6 Division of Hospital Medicine, Department of Medicine, Los Angeles General Medical Center, Los Angeles, California; 7 Department of Emergency Medicine, Los Angeles General Medical Center, Los Angeles, California; 8 Patient and Family Advisory Council, Los Angeles General Medical Center, Los Angeles, California; 9 School of Nursing, University of California, Los Angeles

## Abstract

**Importance:**

Uninsured patients who initiate hemodialysis (HD) typically cannot be placed in outpatient HD centers until their insurance applications are processed. Allowing these patients temporary access to an inpatient HD unit as outpatients could shorten their hospital length of stay (LOS).

**Objective:**

To evaluate the association of hospital LOS with a first-in-state model in which an inpatient HD unit received regulatory program flex approval to provide temporary, transitional outpatient access to uninsured patients.

**Design, Setting, and Participants:**

This quasi-experimental, pre-post quality improvement study was conducted at 1 intervention hospital and 2 control hospitals within the safety net of the Los Angeles County Department of Health Services among uninsured inpatients who newly initiated HD from January 1, 2016, through December 31, 2024.

**Exposure:**

Transitional outpatient HD access to an inpatient unit was implemented in February of 2020 at the Los Angeles General Medical Center (LA General). Control hospitals began to use their emergency departments for outpatient HD access during the intervention period.

**Main Outcomes and Measures:**

The primary outcome measure was the difference in LOS before vs after transitional HD implementation. Secondary outcomes included all-cause 30-day readmission and mortality rates.

**Results:**

Overall, 951 patients were included in this study. In the preintervention period, LA General treated 200 uninsured inpatients newly initiating HD (mean [SD] age, 52.0 [11.7] years; 130 men [65.0%]) vs 241 patients (mean [SD] age, 52.9 [11.1] years; 171 men [71.0%]) in the postintervention period. The mean (SD) LOS at LA General was significantly shorter after vs before the intervention (7.6 [6.6] vs 13.0 [17.5] days; *P* < .001). At control hospitals, 234 patients (mean [SD] age, 52.4 [13.6] years; 164 men [70.1%]) were treated in the preintervention period, and 276 patients (mean [SD] age, 52.7 [12.5] years; 209 men [75.7%]) were treated in the postintervention period. The mean (SD) LOS at control hospitals was significantly shorter after vs before the intervention (9.1 [9.4] vs 12.5 [15.3] days; *P* = .002). Difference-in-difference analysis did not demonstrate a reduction in LOS at LA General vs control hospitals (−2.0 days; *P* = .23). However, run charts demonstrated immediate, sustained reductions in LOS at LA General but variations in LOS after the intervention at the control hospitals, such that the parallel trends assumption for difference-in-difference validity was likely not met.

**Conclusions and Relevance:**

In this quality improvement study, regulatory approval of an inpatient HD unit to provide transitional, outpatient services to uninsured patients newly initiating HD was associated with a significantly reduced LOS. These results suggest that this model could be a viable solution for other hospitals facing similar difficulties with expeditious discharge planning for uninsured patients undergoing HD.

## Key Points

**Question:**

Can hospital length of stay (LOS) be shortened for uninsured inpatients initiating hemodialysis (HD) by allowing them temporary, postdischarge, outpatient access to a first-in-state, regulatorily approved transitional HD unit, until their insurance activates, enabling HD center placement?

**Findings:**

This quality improvement study with 951 participants found that offering postdischarge, transitional outpatient access to an inpatient HD unit shortened hospital LOS by a mean of 5 days for uninsured patients newly initiating HD.

**Meaning:**

This study suggests that offering transitional HD services to uninsured inpatients newly initiating HD, who otherwise could not access outpatient HD until insurance became active, was associated with a significantly reduced hospital LOS.

## Teaser

This quality improvement study evaluates the association of hospital length of stay with a first-in-state model in which an inpatient hemodialysis unit received regulatory approval to provide temporary, transitional outpatient access to uninsured patients.

## Introduction

Patients who initiate hemodialysis (HD) in the inpatient setting are more likely to have multiple comorbidities, poor social support, and/or referral to nephrology late in their course.^1^ Such patients are especially familiar to safety net health systems, which serve the nation’s most vulnerable patients, many of whom are uninsured and have not accessed regular care prior to arriving to the emergency department. After stabilization and initiation of HD, a secure plan for outpatient HD after patients leave the hospital is required to enable hospital discharge. However, access to ambulatory dialysis centers requires insurance. Thus, uninsured patients who newly initiate HD must typically remain in the hospital until their applications for Medicaid or Medicare are processed and approved. This process can take days to weeks.

In response to this challenge, the Los Angeles General Medical Center (LA General), the largest hospital in the Los Angeles County Department of Health Services (LAC DHS) safety net public hospital system, posed a question to regulatory experts with the California Department of Public Health (CDPH): could the hospital’s inpatient HD unit become licensed to provide short-term, transitional, outpatient HD services for uninsured patients as a hospital discharge plan, while the patients’ applications for Medicaid or Medicare were processed? We emphasized that, in the current state, those patients were remaining in the hospital for the sole purpose of receiving HD in the same inpatient HD unit.

LA General worked with CDPH for several months to develop a process that would satisfy all regulatory requirements, and the hospital received approval to launch the program on February 18, 2020. Here, we report on changes in length of stay (LOS) associated with this focused, regulatory program flex approval, which allowed for temporary, transitional outpatient HD in the inpatient unit for up to 10 business days.

## Methods

### Study Design, Setting, and Population

LA General is a county-operated, 676-bed, level I trauma teaching hospital near downtown Los Angeles. It is 1 of 3 acute care public hospitals, along with Harbor-UCLA and Olive View Medical Centers, in the LAC DHS safety net system. The LAC DHS system is the second-largest municipal health system in the US, operating hospitals and a network of public safety net outpatient clinics, but no outpatient HD centers. Patients in the LAC DHS system with end-stage kidney disease (ESKD) receive outpatient HD at private, community centers. Reporting of the analysis of this preintervention and postintervention, quality improvement cohort study followed the Standards for Quality Improvement Reporting Excellence (SQUIRE) reporting guideline. The study was approved as expedited and with a waiver of informed consent by the University of Southern California institutional review board because data were collected for standard operations and evaluated retrospectively with deidentification.

Approximately 20% of LA General inpatients lack insurance at the time they are admitted to the hospital. Thus, we frequently encounter patients newly requiring HD who lack necessary funding to access outpatient HD centers. These patients typically wait in the hospital while their Medicaid or Medicare applications are submitted and processed. In response, LA General entered into discussions with our partners at the CDPH, which licenses health care facilities in California. The LA General request was to establish a transitional HD unit to enable uninsured patients initiating HD to leave the hospital with a solid HD discharge plan, pending processing of their insurance such that outpatient HD centers would accept them. Given the differences in regulatory requirements for inpatient and outpatient HD units, CDPH ultimately authorized a “program flex.” The program flex waived outpatient HD center requirements for our inpatient HD unit on a time-limited, focused basis specifically and only for the purpose of allowing up to 10 business days of outpatient HD access for patients initiating HD during hospitalization who could not otherwise access private outpatient HD centers.

On February 18, 2020, LA General’s newly approved transitional HD unit process went live. We subsequently evaluated the outcomes of patients admitted to the LAC DHS acute care hospitals in the period before (January 16, 2016, to February 17, 2020) and after (February 18, 2020, to December 31, 2024) the process was implemented at LA General. The other 2 LAC DHS acute care hospitals, which did not implement a similar process during the study period, served as controls. However, both control hospitals began using their emergency departments to enable some outpatient HD access during the postintervention period while patients were awaiting insurance processing.

### Data Collection

Data were obtained from the Vizient Clinical Data Base, of which LAC DHS is a partnering institution and to which LAC DHS inputs data extracted from its Cerner electronic health record. Patient demographic data included age, sex, race (Asian, Black, White, or other [option chosen if patients did not self-identify with any of the specified categories]), ethnicity (Hispanic or non-Hispanic origin), and financial codes. Race and ethnicity are collected by all hospitals as a regulatory requirement, self-reported by the patient on admission to the hospital, and entered into the electronic health record by registrational personnel. Clinical data included mortality, length of hospitalization, *Current Procedural Terminology* (*CPT*) and *International Statistical Classification of Diseases, Tenth Revision, Clinical Modification* (*ICD-10-CM*), and *International Statistical Classification of Diseases, Tenth Revision, Procedure Coding System* (*ICD-10-PCS*) codes. Case mix index, defined as the mean of the relative weights of the Medicare Severity Diagnostic Related Groups (MS DRGs) assigned to patients at discharge, was also assessed.

### Patient Inclusion and Exclusion Criteria

**Figure 1.**
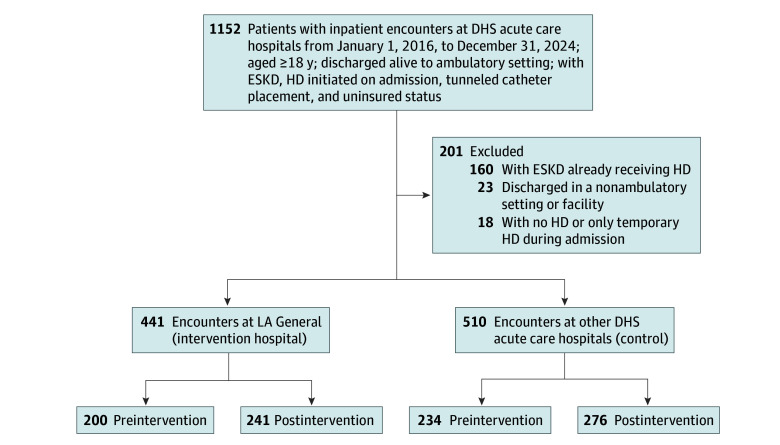
Study Cohort Selection and Inclusion Criteria DHS indicates Department of Health Services; ESKD, end-stage kidney disease; and HD, hemodialysis.

The preintervention cohorts received inpatient care from January 1, 2016, through February 17, 2020. The postintervention cohorts received inpatient care from February 18, 2020, through December 31, 2024. Inclusion criteria were all uninsured patients aged 18 years or older, discharged alive between January 1, 2016, and December 31, 2024, who initiated HD for ESKD during hospital admission (Figure 1). These patients were initially identified by the presence of ESKD *ICD-10-CM* codes, a *CPT* code for tunneled catheter placement, and financial codes representative of uninsured status (Box 1). All patients identified through this query subsequently underwent medical record review to select the final study cohorts.

Box 1. **Diagnosis, Procedure, and Financial Codes Used in Cohort Selection Query**

***ICD-10-CM* codes used to identify ESKD**

N186, Z992, N185, I120, E112, E1121, E1122, E1129

***CPT* code used to identify tunneled catheter placement**

36558

**Financial codes used to identify uninsured patients^a^**

000 (self pay outpatient), 350/351 (Ability To Pay), 402 (hospital presumptive eligibility), 403/406 (Medi-Cal restricted benefits), 407 (Medi-Cal pending district), 423 (Medi-Cal pending other district), 469 (self pay inpatient), 501 (out-of-county/out-of-country)

Abbreviations: *CPT*, *Current Procedural Terminology*; ESKD, end-stage kidney disease; *ICD-10-CM*, *International Statistical Classification of Diseases and Related Health Problems, Tenth Revision, Clinical Modification*.

^a^
Ability To Pay is a county payment program that enables unfunded patients to pay a small proportion of their health care costs so that it is affordable to them; presumptive eligibility applies to unfunded patients who meet Medicaid criteria, such that an application for Medicaid is submitted while they are inpatient, and can retroactively fund the inpatient stay, but does not cover outpatient care until the Medicaid becomes active. Medi-Cal restricted covers only emergency inpatient care and no outpatient care.

After medical record review, patients were included if the review confirmed that they had newly initiated HD and were alive and discharged to their home. Patients were excluded if they were already receiving HD in Los Angeles County at the time of hospital admission, or if they died during hospitalization or were sent to a different hospital, rehabilitation facility, skilled nursing facility, or jail at hospital discharge. Patients were also excluded if they were only temporarily in Los Angeles and were planning to return to a different county, state, or country at hospital discharge.

### Patient Outcomes

The primary outcome was hospital LOS for included patients at LA General before and after the program’s implementation. Secondary outcomes included hospital LOS at LA General compared with the control hospitals, as well as all-cause 30-day hospital readmission and 30-day mortality rates for both LA General and control hospitals, before and after the intervention.

### Statistical Analysis

Continuous variables were compared with the *t* test, after confirming the normality of the data by the Kolmogorov-Smirnov test. Cohort comparisons of proportional data (eg, sex) used the χ^2^ test or the Fisher exact test for sample sizes of 5 or fewer. Difference-in-difference (DiD) estimation was used as a sensitivity analysis to measure the effect of the intervention. Run charts were used to assess quarter to quarter fluctuations in LOS before and after the intervention. All statistical testing was 2-sided, and results were deemed statistically significant at *P* < .05. KyPlot, version 5.0 (Informer Technologies Inc) was used to perform Kolmogorov-Smirnov testing; all other data were analyzed using Stata, version 17.0 (StataCorp LLC).

## Results

### Study Cohort Characteristics

A total of 951 patients were included in the study. At LA General, 200 uninsured patients who newly initiated HD were treated before transitional HD implementation (mean [SD] age, 52.0 [11.7] years; 130 men [65.0%] and 70 women [35.0%]), and 241 patients were treated after transitional HD implementation (mean [SD] age, 52.9 [11.1] years; 171 men [71.0%] and 69 women [28.6%]) (Table 1). Preintervention and postintervention cohorts were similar in age (mean [SD] age, 52.0 [11.7] years vs 52.9 [11.1] years; *P* = .39), sex (35.9% vs 28.6% female; *P* = .25), language (English, 23 [11.5%] vs 32 [13.3%]; Spanish, 170 [85.0%] vs 202 [83.8%]; other language, 7 [3.5%] vs 7 [2.9%]; *P* = .81), and case mix index (MS DRG relative weights, 2.48 [1.54] vs 2.46 [1.60]; *P* = .94). However, the cohorts differed significantly by race (Asian, 8 [4.0%] vs 9 [3.7%]; Black; 4 [2.0%] vs 5 [2.1%]; White, 12 [6.0%] vs 0; other race, 176 [88.0%] vs 227 [94.2%]; *P* = .002) and ethnicity (Hispanic, 168 [84.0%] vs 221 [91.7%]; non-Hispanic, 32 [16.0%] vs 20 [8.3%]; *P* = .01).

**Table 1.** Patient Cohort Characteristics

Characteristic	Intervention hospital			Control hospitals		
	Preintervention period, No. (%)	Postintervention period, No. (%)	*P* value	Preintervention period, No. (%)	Postintervention period, No. (%)	*P* value
Patients	200 (45.4)	241 (54.6)	NA	234 (45.9)	276 (54.1)	NA
Age, mean (SD), y	52.0 (11.7)	52.9 (11.1)	.39	52.4 (13.6)	52.7 (12.5)	.80
Sex						
Female	70 (35.0)	69 (28.6)	.25	70 (29.9)	67 (24.3)	.15
Male	130 (65.0)	171 (71.0)		164 (70.1)	209 (75.7)	
Unknown	0	1 (0.4)		0	0	
Race^a^						
Asian	8 (4.0)	9 (3.7)	.002	8 (3.4)	15 (5.4)	<.001
Black	4 (2.0)	5 (2.1)		12 (5.1)	6 (2.2)	
White	12 (6.0)	0		21 (9.0)	5 (1.8)	
Other^b^	176 (88.0)	227 (94.2)		193 (82.5)	250 (90.6)	
Ethnicity^a^						
Hispanic	168 (84.0)	221 (91.7)	.01	200 (85.5)	240 (87.0)	.63
Non-Hispanic	32 (16.0)	20 (8.3)		34 (14.5)	36 (13.0)	
Language						
English	23 (11.5)	32 (13.3)	.81	42 (17.9)	56 (20.3)	.59
Spanish	170 (85.0)	202 (83.8)		183 (78.2)	213 (77.2)	
Other	7 (3.5)	7 (2.9)		9 (3.8)	7 (2.5)	
Case mix index, mean (SD)	2.48 (1.54)	2.46 (1.60)	.94	2.43 (1.58)	2.51 (1.02)	.45

Abbreviation: NA, not applicable.

^a^ Race and ethnicity are self-reported by patients on admission to the hospital.

^b^ Other race was the option chosen if patients did not self-identify with any of the specified racial categories.

For the control hospitals, 234 patients (mean [SD] age, 52.4 [13.6] years; 164 men [70.1%] and 70 women [29.9%]) were treated during the baseline period, and 276 patients (mean [SD] age, 52.7 [12.5] years; 209 men [75.7%] and 67 women [24.3%]) were treated during the intervention period (Table 1). Age (mean [SD] age, 52.4 [13.6] vs 52.7 [12.5] years; *P* = .80), sex (29.9% vs 24.3% female; *P* = .15), ethnicity (Hispanic, 200 [85.5%] vs 240 [87.0%]; non-Hispanic, 34 [14.5%] vs 36 [13.0%]; *P* = .63), language (English, 42 [17.9%] vs 56 [20.3%]; Spanish, 183 [78.2%] vs 213 [77.2%]; other language, 9 [3.8%] vs 7 [2.5%]; *P* = .59), and case mix index (mean [SD] MS DRG relative weights, 2.43 [1.58] vs 2.51 [1.02]; *P* = .45), were similar for the preintervention and postintervention cohorts. The only significant difference observed between the preintervention and postintervention cohorts was race (Asian, 8 [3.4%] vs 15 [5.4%]; Black; 12 [5.1%] vs 6 [2.2%]; White, 21 [9.0%] vs 5 [1.8%]; other race, 193 [82.5%] vs 250 [90.6%]; *P* < .001).

### Patient Outcomes

**Figure 2.**
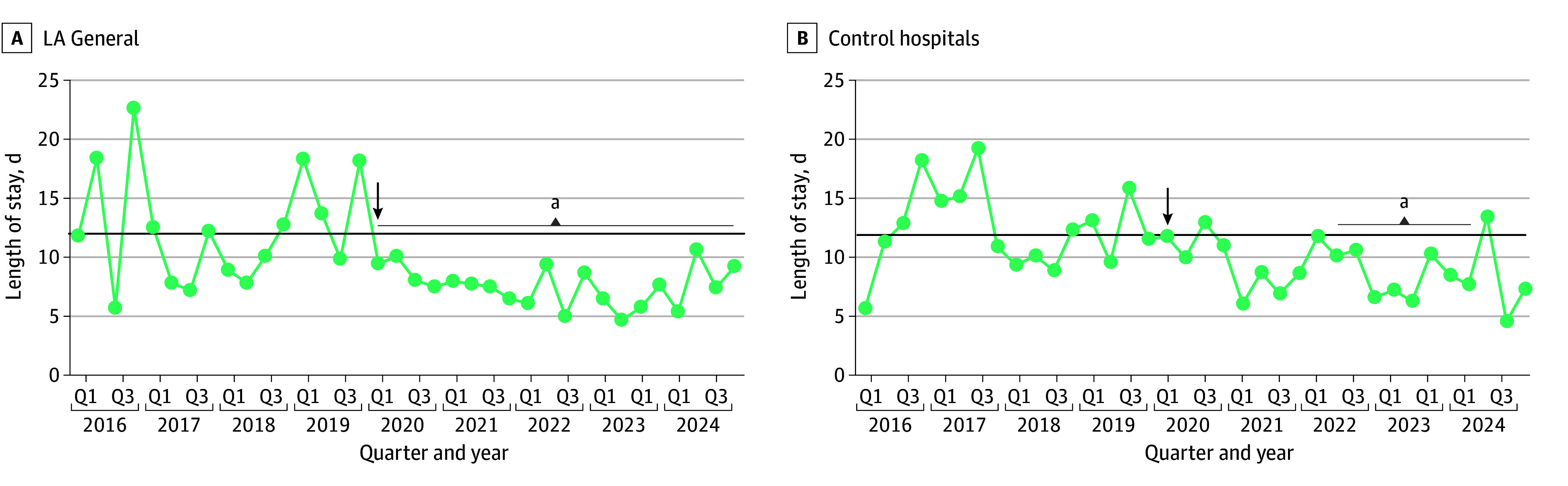
Run Charts for Los Angeles General Medical Center (LA General) and Control Hospitals Spanning the Preintervention and Postintervention Periods The arrows indicate the quarter the intervention began at LA General. ^a^Statistically significant runs, as indicated by at least 6 consecutive data points above or below the median line.^2^

At LA General, patients had significantly shorter mean (SD) LOS after compared with before the intervention (7.6 [6.6] vs 13.0 [17.5] days; *P* < .001) (Table 2). Control hospitals also experienced a significant reduction in observed LOS before compared with after the intervention (9.1 [9.4] vs 12.5 [15.3] days; *P* = .002). As a sensitivity analysis, the mean (SE) DiD estimation in observed LOS was −2.0 (1.7) days (*P* = .23) between intervention and control hospitals. However, we also analyzed run charts to assess variability in LOS from quarter to quarter. LA General experienced an immediate statistical run below the median line in LOS the same quarter that the intervention began and sustained that reduction for 20 consecutive quarters, throughout the entire 4-year intervention period (Figure 2).^2^ In contrast, the control hospitals experienced more variability from quarter to quarter in LOS, such that there was a statistical run for 8 consecutive quarters from the second quarter of 2022 through the first quarter of 2024.

**Table 2.** Observed Length of Stay, Difference in Differences, and Balancing Measures

Outcome	Intervention hospital			Control hospitals		
	Preintervention period	Postintervention period	*P* value	Preintervention period	Postintervention period	*P* value
Length of stay, mean (SD), d^a^	13.0 (17.5)	7.6 (6.6)	<.001	12.5 (15.3)	9.1 (9.4)	.002
Balancing measures, % (No/total No.)						
30-d Readmission rate	13.0 (26/200)	15.8 (38/241)	.41	10.7 (25/234)	16.3 (45/276)	.07
30-d Mortality rate	0 (0/200)	1.2 (3/241)	.11	0 (0/234)	1.1 (3/276)	.11

^a^ The difference-in-difference estimation for the primary outcome of mean (SE) length of stay is −2.0 (1.7) days (*P* = .23).

Finally, as balancing safety measures, no significant differences were observed after vs before the intervention at either LA General or the control hospitals in all-cause 30-day readmission rates (LA General, 15.8% [38 of 241] after vs 13.0% [26 of 200] before the intervention; *P* = .41; controls, 16.3% [45 of 276] after vs 10.7% [25 of 234] before intervention; *P* = .07) or all-cause 30-day mortality rates (LA General, 1.2% [3 of 241] after vs 0.0% before the intervention; *P* = .11; controls, 1.1% [3 of 276] after vs 0.0% before the intervention; *P* = .11) (Table 2).

## Discussion

Historically, regulations have strictly prohibited the use of inpatient units to provide outpatient HD services. According to the CDPH, LA General was the first hospital in California to request regulatory approval from CDPH to set up a transitional HD unit to help uninsured patients initiating HD leave the hospital before their insurance applications were approved. Here, we report that this novel intervention was associated with reduced hospital LOS for these patients while they awaited Medicaid or Medicare activation, so they could then transition to permanent outpatient HD facilities.

### Strengths and Limitations

Our study has several strengths. The transitional HD unit intervention was a regulatory innovation, reflecting a partnership between the LA County DHS and CDPH. Allowing a limited-scope, targeted program flex for the inpatient HD unit, after meeting appropriate clinical and regulatory stipulations, advanced the triple aim of health care. First, it helped us significantly reduce the time patients spent in the hospital, a safety and quality goal.^3^ Second, this was done with existing resources, thereby reducing the cost of care—in this case, the hospital expedited discharge without the system investing unnecessarily in its own outpatient HD centers. Third, the clinical problem and intervention also called attention to patient experience, as uninsured patients can often least afford to remain in the hospital. One of our coauthors (M.V.), who is a retired emergency medical technician and a member of the LA General Patient and Family Advisory Council, happened to be hospitalized during the postintervention period at one of the control hospitals, specifically for initiation of HD. We have asked him to tell his story to illustrate from a patient perspective the importance of this transitional HD innovation (Box 2).

Box 2. **MV’s Story**

I was admitted to one of the control hospitals in Los Angeles with complications of end-stage kidney failure, and ultimately had a catheter placed and was started on hemodialysis.
I was residing in an assisted living facility. During my stay in the hospital, I received a call from animal control indicating that they would be taking custody of my dogs for abandonment. I explained my situation to the animal control officers, who allowed my dogs to remain temporarily as long as a family member picked them up. I felt incredibly anxious, and I pleaded to be discharged, but I still did not have outpatient hemodialysis placement. Social services informed me that they were rarely able to place a new outpatient hemodialysis patient within 10 days. My anxiety only increased.
Fortunately, hemodialysis placement happened sooner than expected. Nevertheless, I was in hospital for multiple days only for the purpose of outpatient hemodialysis placement, and during that time I was at risk not only of losing my spot in assisted living, but losing my beloved therapy dogs.

Our study also has several important limitations. First, the quasi-experimental pre-post design, while preferable to a noninterventional retrospective study, is still potentially prone to confounding, such as by secular trends in care other than the intervention. For example, the intervention occurred right before the COVID-19 pandemic began in California. However, the COVID-19 pandemic would likely have prolonged LOS for such patients after the intervention, because HD centers were careful in requiring negative testing before booking patient appointments. Because patients who died in the hospital were excluded, COVID-19 would not have shortened LOS due to inpatient deaths. The intervention facilitated shorter lengths of stay despite COVID-19. Although the pre-post cohorts were generally well matched, there were some differences in demographics, which underscored the potential for confounding. Nevertheless, the differences were small and of unclear relevance to LOS.

There are also limitations in our use, findings, and interpretation of the sensitivity analysis completed using a DiD estimation. DiD estimations can be useful for pre-post intervention studies; however, the control hospitals modified practices in the postintervention period, which could have violated the parallel trends assumption that is required for a DiD analysis to be accurate.^4^ Run charts demonstrated that, while the intervention hospital (LA General) exhibited little variation from quarter to quarter after the intervention, the control hospitals did exhibit significant variation from quarter to quarter, which resulted in variation in LOS between LA General and control hospitals that likely violated the parallel trends assumption.

## Conclusions

In this pre-post quality improvement study, we found that implementation of a novel transitional HD unit, with regulatory program flex approval by CDPH, was associated with a significant reduction in hospital LOS for uninsured patients newly initiating HD. For this process to work, some payer mechanism is required to eventually become active to enable the patient to switch to HD in an outpatient facility. M.V.’s story (Box 2) underscores the impact this can have, particularly on vulnerable patients who are uninsured or have unstable housing or financial situations. This innovative program was developed in collaboration with state regulators and was readily implemented at a large, public, teaching, safety net hospital in California. These results suggest that such a program is a viable solution for other hospitals facing similar difficulties with expeditious discharge planning for uninsured patients undergoing HD.

## Notes

Supplement.
Data Sharing Statement
