# Supplementary material for: Provision of Temporary Access to Inpatient Hemodialysis to Uninsured Patients Initiating Hemodialysis
Source: JAMA Netw Open. 2025 Nov 18;8(11):e2544295. doi: 10.1001/jamanetworkopen.2025.44295 (PMC12628098; doi:10.1001/jamanetworkopen.2025.44295)
Supplement: Supplement. — Data Sharing Statement [file jamanetwopen-e2544295-s001.pdf]

# Data Sharing Statement

Banerjee. Provision of Temporary Access to Inpatient Hemodialysis to Uninsured Patients Initiating Hemodialysis. *JAMA Netw Open*. Published November 18, 2025. doi:10.1001/jamanetworkopen.2025.44295

**Data**
**Data available:** No

**Additional Information**
**Explanation for why data not available:** This was a QI study and the IRB approval was Exempt due to collecting data for QI purposes as part of hospital operations.
